# Intrahepatic cholangiocarcinoma patients without indications of lymph node metastasis not benefit from lymph node dissection

**DOI:** 10.18632/oncotarget.22852

**Published:** 2017-12-01

**Authors:** Jie Hu, Fei-Yu Chen, Kai-Qian Zhou, Cheng Zhou, Ya Cao, Hui-Chuan Sun, Jia Fan, Jian Zhou, Zheng Wang

**Affiliations:** ^1^ Liver Cancer Institute, Zhongshan Hospital, Fudan University, Shanghai, Key Laboratory of Carcinogenesis and Cancer Invasion, Fudan University, Ministry of Education, Shanghai, China; ^2^ Cancer Research Institute, Xiangya School of Medicine, Central South University, Hunan, China; ^3^ State Key Laboratory of Genetic Engineering, Fudan University, Shanghai, China; ^4^ Shanghai Key Laboratory of Organ Transplantation, Zhongshan Hospital, Fudan University, Shanghai, China; ^5^ Institute of Biomedical Sciences, Fudan University, Shanghai, China

**Keywords:** intrahepatic cholangiocarcinoma, lymph node metastasis, lymph node dissection, prognosis

## Abstract

Background: To investigate the necessity of routine lymph node dissection (LND) in intrahepatic cholangiocarcinoma (ICC) patients without indications of lymph node metastasis (LNM) preoperatively. Methods: 422 consecutive ICC patients who undergone curative resection from January 2009 to December 2014 were enrolled and categorized as two groups (hepatectomy only or hepatectomy plus LND). Clinicopathologic data was compared between the groups by χ^2^ or Fisher’s exact test. Overall survival (OS) and recurrence-free survival (RFS) were calculated by the Kaplan–Meier method and differences were analyzed using the log-rank test. Cox regression model was adopted for multivariable analysis. Results: The median OS time of all 422 patients was 41.4 months. One-, 3-, and 5-year OS was 67%, 47%, and 35%, respectively. A total of 73 patients had undergone curative resection combined with LND, of whom 20.5% (15/73) were confirmed lymph node positive pathologically. The clinicopathologic characteristics between LND and control groups showed no significant differences. Of the 422 patients, 271 patients had recurrence. The recurrence rates were 65.8% for the LND group and 63.9% for the non-LND group. Survival analysis revealed that, neither the OS (LND vs. non-LND: 32.2 months vs. 46.2 months; p = 0.16) nor the RFS (LND vs. non-LND: 23.1 months vs. 17.0 months; p = 0.09) had significant difference. Multivariate analysis revealed that tumor size, tumor number, carbohydrate antigen19-9, carcinoembryonic antigen, and gamma-glutamyl transpeptidase were independent predictive factors for OS and RFS. Conclusion: Routine LND may not improve survival in resectable ICC patients with negative LNM diagnosis before operation.

## INTRODUCTION

Intrahepatic cholangiocarcinoma (ICC) is the second most common primary liver malignancy following hepatocellular carcinoma (HCC) [1]. Incidence of ICC has been steadily increasing for the past three decades worldwide [2], without much improvement in mortality [3–5]. Unlike HCC which arises in the background of chronic liver disease, ICC often occurs in people with no definite liver disease. Therefore, prevention or screening strategies seem get no way to start. The poor prognosis of ICC is of particular concern. Although resection is considered the only choice of curative treatment for ICC, the prognosis of ICC is still unsatisfactory, regardless of aggressive surgical treatment [6, 7].

Several clinicopathological factors, including tumor number, vascular invasion, distal metastasis, as well as some other clinical parameters, have been evaluated as indicators for survival. Among the parameters studied, lymph node metastasis (LNM) is one of the most relevant factors [8–10]. Patients with positive LNM have unfavorable prognosis [11–14]. However, the necessity of routine lymphadenectomy remains controversial, especially for patients evaluated as negative for lymph node involvement before operation. Some researchers have suggested routine lymph node dissection (LND) for decreasing locoregional recurrence and optimizing pathologic staging [8, 9]. Others have recommended against performing LND routinely, doubting its value for prolonging patient survival [15]. However, all of those studies did not describe the preoperative diagnosis of lymph node involvement when focused on evaluation of the impact of LND. In this study, we analyzed clinicopathological data from 422 consecutive ICC patients to assess the impact of LND on patient’s survival, to clarify the utility of routine LND in surgical treatment of ICC patients without LNM.

## RESULTS

### Patient and tumor characteristics

**Figure 1:**
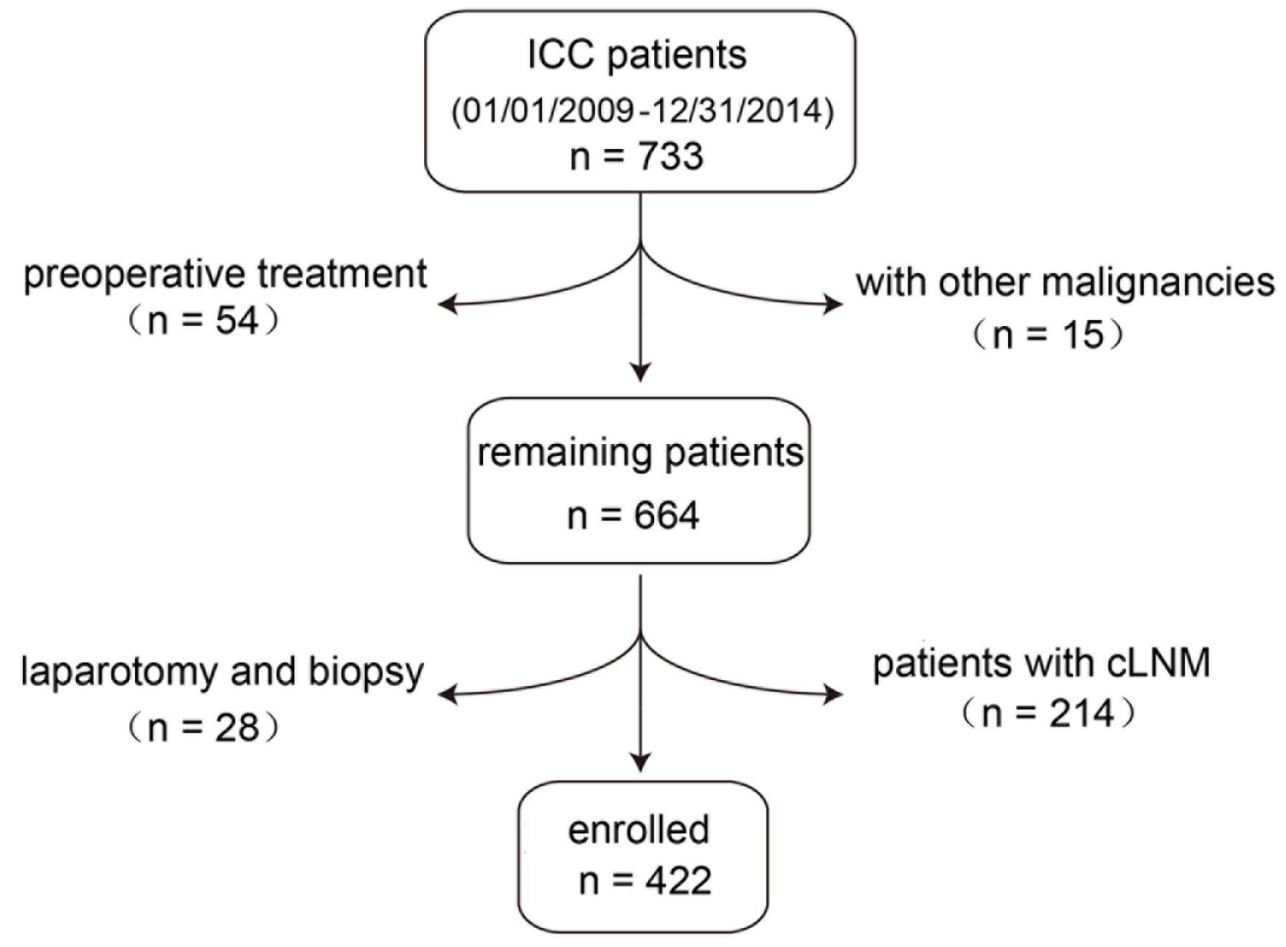
Schematic of process for enrolling patients A total of 733 patients were diagnosed with ICC and confirmed pathologically from January 2009 to December 2014. We excluded 54 patients with preoperative treatment; 15 patients were excluded for other accompanying malignancies; 28 patients were excluded because they only received a laparotomy and biopsy. Evidence of LNM, either by intraoperative palpation or by positive imaging examination before operation, was defined as clinical Lymph Node Metastasis (cLNM). After evaluation, 422 patients were finally enrolled in the study.

A total of 733 patients were diagnosed with ICC and confirmed pathologically at the Liver Surgery Department, Zhongshan Hospital, Fudan University (Shanghai, China) from January 2009 to December 2014. Of these 733 patients, 311 did not meet the entry criteria and were excluded. Among them, 54 were excluded for receiving other treatments preoperatively, namely liver resection, transarterial chemoembolization and radiofrequency ablation; 15 patients were excluded due to the presence of other primary malignancies concurrently (including patients with ICC combined with other primary liver malignancies); 28 patients were excluded because they only received a laparotomy and biopsy; and 214 patients with cLNM-positive were also excluded. A total of 422 ICC patients with cLNM-negative met the inclusion criteria specified for this study (Figure 1). The demographic and clinicopathologic data for the enrolled patients are summarized in Table 1 and the data for LND patients are in Supplementary Table 1. Solitary tumor was present in a majority of patients (n = 335; 79.4%) and the median tumor size was 5.2 cm (range, 0.8 to 18.0 cm). After surgical resection, 169 patients (40.0%) received adjunctive treatments, including adjuvant chemotherapy and radiation therapy.

**Table 1:** Clinical characteristics of enrolled patients

Variable	All Patients (n = 422)		LND (n = 73)		non-LND (n = 349)		p
	NO.	%	NO.	%	NO.	%	
**Age**, years							0.12
Median	60		61		59		
Range	27-80		40-74		27-80		
**Sex**							0.15
Male	177	41.9	37	50.7	140	40.1	
Female	245	58.1	36	49.3	209	59.9	
**Tumor number**							0.24
Solitary	337	79.8	62	84.9	275	78.8	
Multiple	85	20.2	11	15.1	74	21.2	
**Tumor size**, cm							0.13
Median	5.3		6.0		5.0		
Range	0.8-18.0		1.5-12.0		0.8-18.0		
**Vessel invasion**							0.91
Vascular	14	3.3	1	1.4	13	3.7	
Biliary	5	1.2	1	1.4	4	1.1	
vascular & biliary	2	0.2	0	0.0	2	0.6	
**Type of liver resection**							0.23
Hemihepatectomy	307	72.7	49	67.1	258	73.9	
Extended hemihepatectomy	68	16.1	10	13.7	58	16.6	
Central hepatectomy	44	10.4	13	17.8	31	8.9	
Unknown	3	0.7	1	1.4	2	0.6	
**T stage**							0.70
T1	320	75.8	59	80.8	261	74.8	
T2							
T2a	11	2.6	2	2.7	9	2.6	
T2b	84	19.9	11	15.1	73	20.9	
T4	7	1.7	1	1.4	6	1.7	
**Adjunctive treatment**							0.32
Yes	169	40.0	33	45.2	136	39.0	
No	253	60.0	40	54.8	213	61.0	
**Postoperative hospital stay**, days							0.03
Average	8.9		9.8		8.7		
Range	3-37		4-35		3-37		

LND, lymph node dissection.

In terms of hepatic resection types, the majority of patients received hepatic resection that was no more than a hemihepatectomy (n = 307; 72.7%), and others received extended hepatectomy (n = 68; 16.1%) or central hepatectomy (n = 44; 10.4%). Vessel invasion was observed in 20 patients (4.7%). Among those with vessel invasion, 14 patients (3.3%) had vascular invasion, 5 patients (1.2%) had biliary invasion, whereas 1 patient (0.2%) had both.

### Lymphadenectomy and prognosis

Among the 422 patients with cLNM-negative ICC who were enrolled in this study, 73 (17.3%) had undergone lymphadenectomy (categorized as the LND group). To explore whether routine LND would benefit ICC patients with cLNM-negative, we first compared the clinical characteristics between the LND and non-LND groups. We found that there was no statistically significant difference in the clinical characteristics analyzed between the two groups (Table 1). However, LND group have longer hospital stay after operation (9.8 day) than that of Non-LND group (8.7 day, p = 0.03).

**Figure 2:**
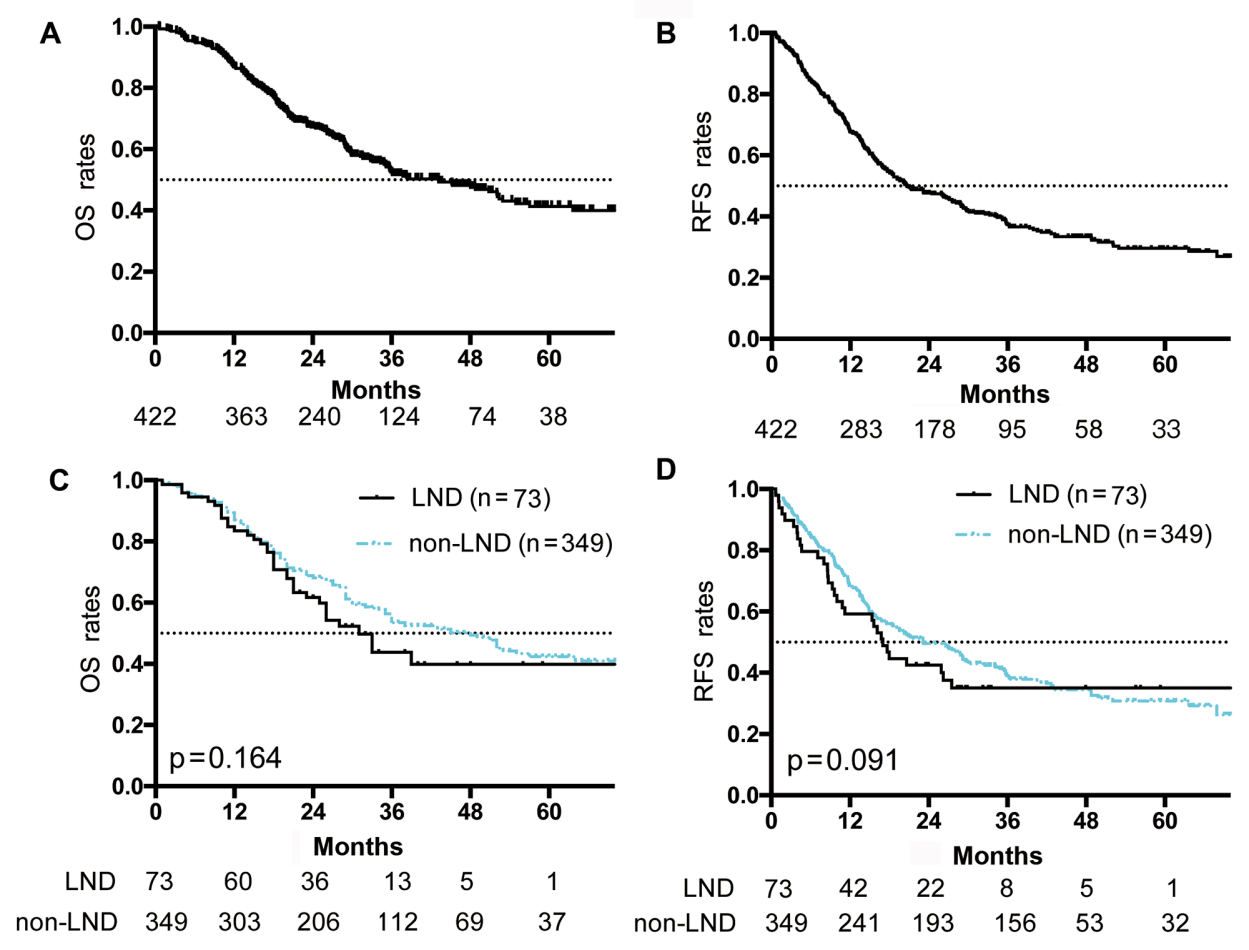
OS and RFS curves of ICC patients without cLNM **(A)** OS curve of all patients. **(B)** RFS curve of all patients. **(C)** OS curves of patients in the lymph node dissection (LND) and non-LND groups. There was no significant survival difference between the two groups (p = 0.16). **(D)** RFS curves of patients in LND and non-LND groups. There was no significant survival difference between the two groups (p = 0.09). Numbers below the graphs show the number of remaining patients at the time point.

The median survival of the LND and non-LND groups was 32.2 and 46.2 months, respectively (p = 0.16). One-, 3-, and 5-year OS rates in the LND group were 64%, 35%, and 35%, respectively, vs. 68%, 49%, and 35%, respectively, in the non-LND group. There was no significant difference in OS (p = 0.16) between the LND and non-LND groups (Figure 2C, Table 2). Of the 422 patients, 271 patients had recurrence. The recurrence rates were 65.8% (n = 48) for the LND group and 63.9% (n = 223) for the non-LND group. There was no significant difference in RFS between the LND and non-LND groups (p = 0.09; Figure 2D). LND was not a predictive factor for RFS in univariate analysis (Table 3).

**Table 2:** Univariate and multivariate analysis of prognostic factors for OS

Prognostic Factor	Univariate		Multivariate	
	HR(95%CI)	P	HR	p
Tumor size (≤2 cm vs. >2 cm)	1.85(1.441-2.366)	0.04	1.51(1.166-1.961)	0.02
Tumor number (single vs. multiple)	2.54(1.873-3.432)	<0.001	2.45(1.780-3.371)	<0.001
CA19-9 (≤37 U/L vs. >37 U/L)	2.18(1.627-2.912)	<0.001	1.61(1.181-2.210)	0.03
CEA (≤5 μg/L vs. >5 μg/L)	2.29(1.687-3.110)	<0.001	1.64(1.186-2.264)	0.03
GGT (≤50 U vs. >50 U)	2.11(1.581-2.828)	<0.001	1.42(1.043-1.949)	0.04
Adjunctive therapy (yes vs. no)	0.85(0.637-1.129)	0.26		
Vessel invasion (yes vs. no)	1.17(0.782-1.747)	0.72		
ALT (≤41 U vs. >41 U)	1.29(0.906-1.817)	0.16		
AST (≤38 U vs. >38 U)	1.33(0.946-1.875)	0.10		
TB (≤17.1 μmol/L vs. >17.1 μmol/L)	0.94(0.522-1.679)	0.83		
DB (≤7 μmol/L vs. >7 μmol/L)	1.21(0.799-1.825)	0.37		
Ascites (yes vs. no)	1.37(0.606-3.081)	0.45		
LND (yes vs. no)	1.29(0.900-1.859)	0.17		

OS, overall survival; HR, Hazards ratio; CA19-9, carbohydrate antigen 19-9; CEA, carcinoembryonic antigen; GGT, gamma-glutamyl transferase; ALT, alanine aminotransferase; AST, aspartate transaminase; T, total bilirubin; DB, direct bilirubin; LND, lymph node dissection.

**Table 3:** Univariate and multivariate analysis of prognostic factors for RFS

Prognostic Factor	Univariate		Multivariate	
	HR(95%CI)	p	HR(95%CI)	p
Tumor size (≤2 cm vs. >2 cm)	1.60(1.182-2.176)	0.002	1.45(1.067-1.968)	0.02
Tumor number (single vs. multiple)	2.08(1.582-2.721)	<0.001	1.90(1.425-2.533)	<0.001
CA19-9 (≤37 U/L vs. >37 U/L)	1.73(1.357-2.214)	<0.001	1.26(0.960-1.663)	0.10
CEA (≤5 μg/L vs. >5 μg/L)	2.08(1.587-2.722)	<0.001	1.69(1.266-2.261)	<0.001
GGT (≤50 U vs. >50 U)	1.84(1.435-2.354)	<0.001	1.47(1.124-1.914)	0.01
Adjunctive therapy (yes vs. no)	0.65(0.509-0.823)	<0.001	0.65(0.503-0.828)	0.001
Vessel invasion (yes vs. no)	1.52(0.867-2.424)	0.008		
ALT (≤41 U vs. >41 U)	1.41(1.046-1.888)	0.02		
AST (≤38 U vs. >38 U)	1.36(1.015-1.825)	0.04		
TB (≤17.1 μmol/L vs. >17.1 μmol/L)	0.98(0.599-1.601)	0.94		
DB (≤7 μmol/L vs. >7 μmol/L)	1.09(0.750-1.570)	0.66		
Ascites (yes vs. no)	1.65(0.818-3.343)	0.16		
LND (yes vs. no)	1.31(0.957-1.790)	0.09		

RFS, recurrence-free survival; HR, Hazards ratio; CA19-9, carbohydrate antigen 19-9; CEA, carcinoembryonic antigen; GGT, gamma-glutamyl transferase; ALT, alanine aminotransferase; AST, aspartate transaminase; T, total bilirubin; DB, direct bilirubin; LND, lymph node dissection.

Even after stratification, for patients with solitary tumor and negative vessel invasion (n = 315), LND patients (n = 58) showed no significant difference in OS (p = 0.15) or RFS (p = 0.07), when compared with non-LND patients (n = 257, Supplementary Figure 1).

**Figure 3:**
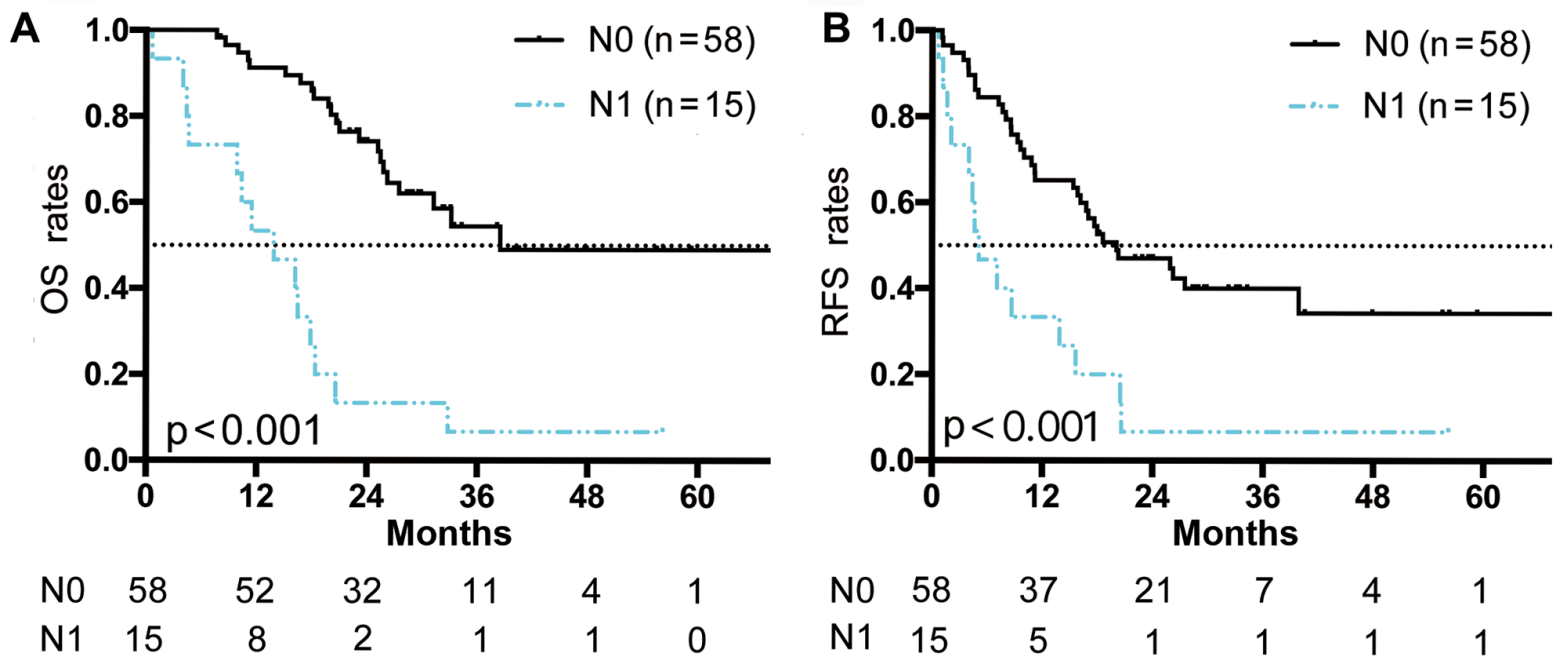
OS and RFS curves of patients who underwent LND **(A)** OS curves of patients who received LND and had a pathological evaluation of their lymph nodes. **(B)** RFS curves of patients who received LND and had a pathological evaluation of their lymph nodes. There was significant survival difference between patients with N0 status and those with N1 status (p < 0.001, respectively). Numbers below the graphs show the number of remaining patients at the time point.

In the LND group (n = 73), 15 patients (20.5%) had LNM, confirmed by pathologic analysis (pathologically-confirmed lymph node metastasis [pLNM]). The presence of N1 status (pLNM) significantly affected OS and RFS. Patients with N0 status showed significantly longer OS time than those with N1 status, with a median survival of 41.5 months vs. 13.0 months (p < 0.001; Figure 3).

**Figure 4:**
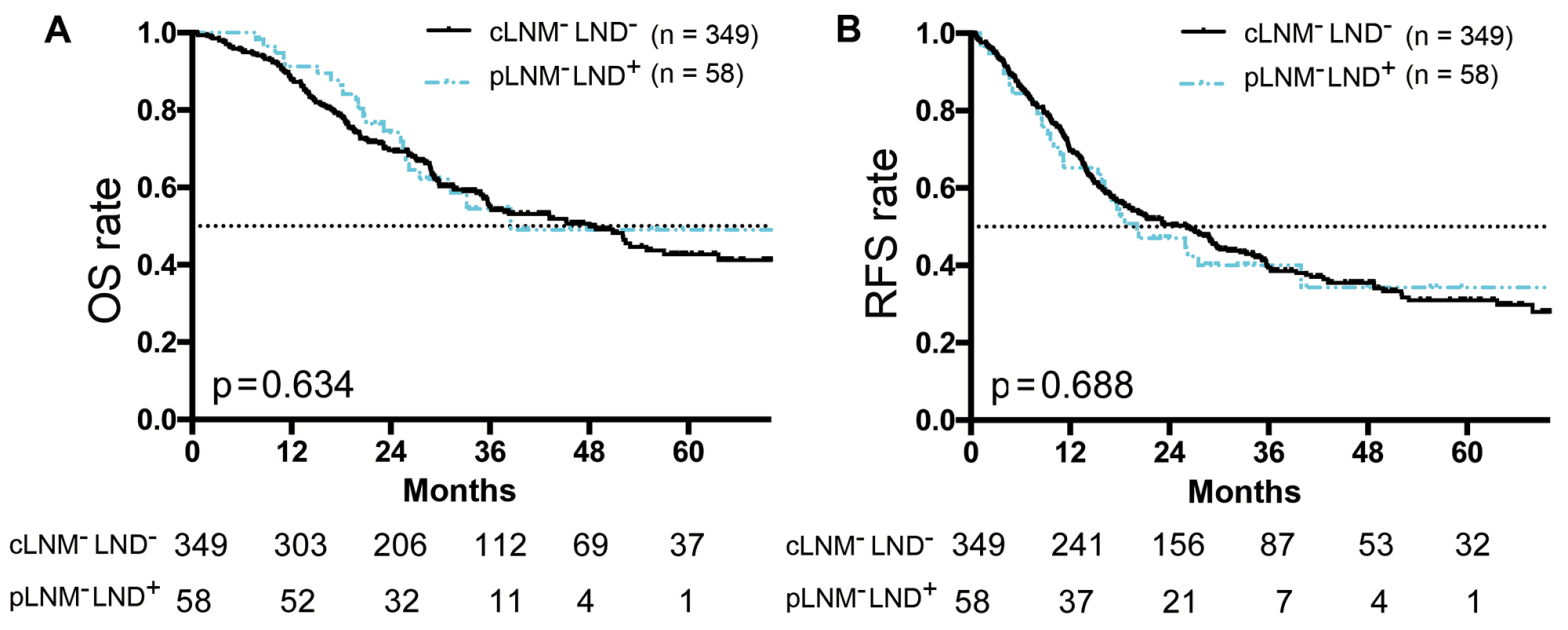
Survival curves of cLNM-neagtive ICC patients (without LND) or pLNM-negative ICC patients (with LND) **(A)** OS curves of non-LND patients with cLNM-negative ICC or LND patients with pLNM-negative ICC. There was no significant survival difference between the two groups (p = 0.63). **(B)** RFS curves of patients with cLNM-negative ICC (without LND) or pLNM-negative ICC (with LND). There was no significant survival difference between the two groups (p = 0.69). Numbers below the graphs show the number of remaining patients at the time point. “**cLNM**^**-**^**LND**^**-**^” represents cLNM-negative patients without LND. “**pLNM**^**-**^**LND**^**+**^” represents patients who underwent LND and were pathologically confirmed as negative for LNM.

We then analyzed the surgical outcomes, comparing the survival time of pLNM-negative patients (n = 58, underwent LND) with those of cLNM-negative patients (n = 349, without LND). The results suggested LND had no significant effect on improving OS (median survival, 41.5 months vs. 46.2 months; p = 0.634; Figure 4A) or RFS (median RFS, 26.1 months vs. 20.1 months; p = 0.688; Figure 4B).

**Figure 5:**
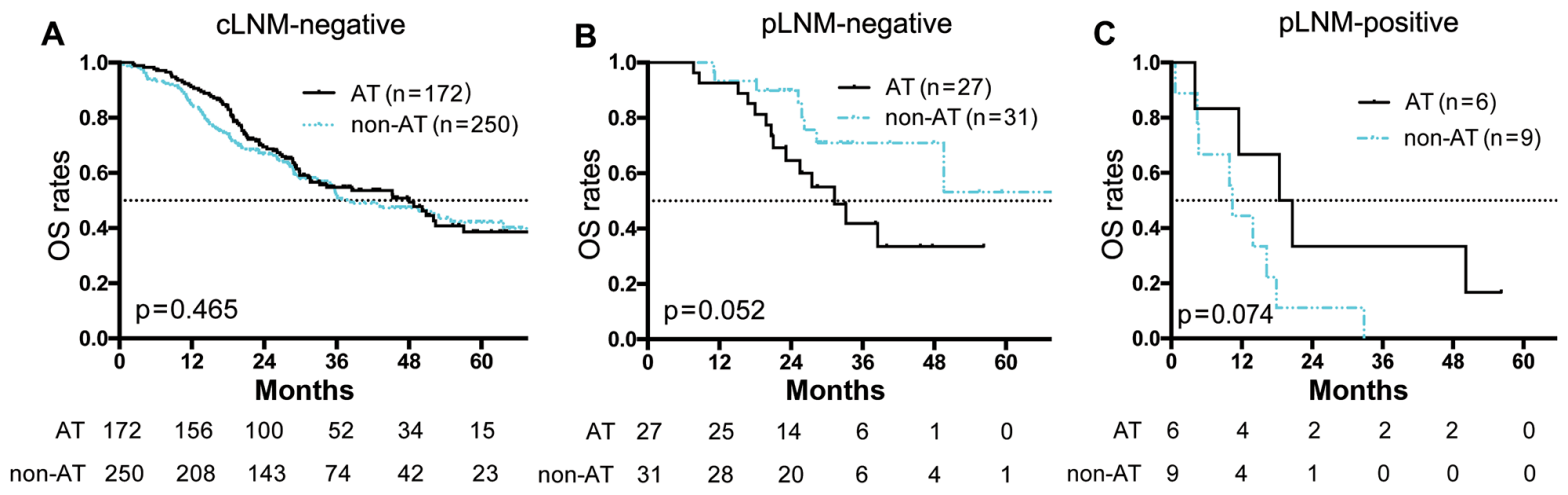
OS curve of ICC patients in adjunctive or non-adjunctive therapy groups **(A)** OS curves of cLNM-negative patients that with adjunctive therapy (AT) or without adjunctive therapy (non-AT). There was no significant difference between the two groups (p = 0.47). **(B)** OS curves of pLNM-negative patients that with AT or with non-AT. There was no significant difference between the two groups (p = 0.052). **(C)** OS curves of pLNM-positive patients that with AT or with non-AT. There was no significant difference between the two groups (p = 0.07). Numbers below the graphs show the number of remaining patients at the time point.

To evaluate whether LND is necessary to ascertain the staging and therefore, set a postoperative treatment plan, we analyzed the value of adjunctive treatment (chemotherapy or radiotherapy) after surgery. Regretfully, postoperative adjunctive treatment did not improve OS in patients with cLNM-negative ICC (n = 422; p = 0.47; Figure 5A) or in patients with pLNM-negative ICC (n = 58; p = 0.052; Figure 5B). Even for patients with pLNM-positive ICC, adjunctive treatment showed no significant impact on OS (n=15, p = 0.07; Figure 5C)

### Prognostic factors

The median OS time of all 422 patients after surgical resection of ICC was 41.37 months. One-, 3-, and 5-year OS was 67%, 47%, and 35%, respectively (Figure 2A and the RFS was shown in Figure 2B). Univariate analysis showed that tumor number (hazards ratio [HR], 2.54; p < 0.001; Table 2) was one of the most significant factors that influenced prognosis. Median survival time for patients with solitary ICC was 56.2 months, compared with 22.0 months for those with multiple ICC lesions (p < 0.001). Other factors associated with survival were tumor size (HR: 1.85; p = 0.04), CA19-9 (HR: 2.18; p < 0.001), CEA (HR: 2.29; p < 0.001), and GGT (HR: 2.11; p < 0.001). On multivariate analysis, factors associated with poor OS were tumor number (HR: 2.45; p < 0.001), tumor size (HR: 1.51; p = 0.02), CA19-9 (HR: 1.61; p = 0.03), CEA (HR: 1.64; p = 0.003), and GGT (HR: 1.42; p = 0.04).

For RFS, the prognostic factors on univariate analysis were tumor number (HR: 2.08; p < 0.001), tumor size (HR: 1.60; p = 0.002), CA19-9 (HR: 1.73; p < 0.001), CEA (HR: 2.08; p < 0.001), GGT (HR: 1.84; p < 0.001), adjunctive therapy (HR: 0.65; p < 0.001), vessel invasion (HR: 1.52; p < 0.001), alanine aminotransferase (ALT, HR: 1.41; p = 0.02), and aspartate aminotransferase (AST, HR: 1.36; p = 0.04). On multivariate analysis, factors associated with poor prognosis were tumor number (HR: 1.90; p <0.001), tumor size (HR: 1.45; p = 0.02), CEA (HR: 1.69; p <0.001), GGT (HR: 1.47; p = 0.01) and adjunctive therapy (HR: 0.65; p = 0.001).

## DISCUSSION

ICC is a cancer characterized by low incidence but high mortality, and the morbidity is even still increasing worldwide. ICC presents higher probability of local LNM than HCC, and the 5-year survival rate of ICC patients is lower when compared with that of HCC patients. Several studies have reported prognostic factors for ICC, with LNM confirmed to be one of the most significant independent indicators [8–10]. In the present study, patients with pLNM indeed had worse outcome even after LND. Although increasing numbers of researchers accept that LNM strongly influences patient survival, the beneficial effect of prophylactic LND on survival remains controversial, especially when treating patients evaluated as negative lymph node involvement before operation.

Many studies supporting prophylactic LND have been reported [9, 10, 15–18]. However, these studies did not show convincing evidence for the beneficial effects of prophylactic LND in patients with cLNM-negative ICC. In our study, LND did not yield a survival benefit for patients with cLNM-negative ICC. The LND group showed similar survival rates and time, regardless of the ending time points (one-, 3-, or 5-year OS). To exclude the effect of other risk factors on survival, we performed the analysis after stratification (solitary tumor without vessel invasion). However, the results still indicated no statistical difference in OS between LND and non-LND patients. We tentatively compared pLNM-negative patients (all underwent LND) with cLNM-negative patients (did not undergo LND), the cLNM-negative patients might include a subgroup of pLNM-postive ICC, which would potentially decrease the OS and RFS. However, we found the survival showed no significance difference between the two groups, implying this subgroup (cLNM-negative but pLNM-postive patients) is small and was insufficient to decrease the survival of overall group. Besides, two other studies have also recommended against LND, citing an absence of survival impact of LND in patients with LNM [6, 11]. Therefore, LND may not improve the OS in cLNM-negative patients.

The high mortality of ICC is associated with high risk of disease recurrence. Considering that lymphadenectomy might not improve the OS in patients with cLNM-negative ICC, we then evaluated whether LND could slow tumor recurrence. However, in the present study, LND was not an indicator for tumor recurrence. Therefore, it seems that the benefit of LND is very limited, in that it neither improves the OS nor attenuates tumor recurrence of patients with ICC, especially those with cLNM-negative ICC.

For patients with cLNM-negative, but pLNM-positive ICC, LND seemed to bring additional benefits for their survivals. Therefore, researchers supporting routine LND argued that preoperative imaging assessment lacks accuracy [15, 19, 20]. However, in this study, only 20% (15/73) cLNM-negative patients were finally proved LNM pathologically. Most of cLNM-negative patients (80%) showed consistent diagnosis after final pathologic evaluations. And we found cLNM-negative ICC patients showed almost the same survival rate when compared with pLNM-negative ICC patients, this result similar with literature report [21, 22]. Besides, with the development of imaging system, the accuracy of LNM detection by preoperative imaging examination has been improving in recent years [23, 24]. Enhanced CT or PET-CT could reach nearly 99% negative predictive value in patients without LNM [25]. Along with routine intraoperative assessment (such as palpation), the accuracy of clinical diagnosis of LNM has been increased and the demand of LND to exclude the false negative of cLNM diagnosis would be diminishing.

Another reason in support of performing LND is that it provides a means for accurate staging and setting postoperative treatment plans [26, 27]. Systemic chemotherapy (gemcitabine based) and radiotherapy are frequently used adjuvant treatment after curative resection [28]. However, these therapies lack of evidence-based validation from phase 3 clinical trials in ICC patients. After analyzed the prognosis value of adjunctive treatment in this study, we found postoperative adjunctive treatment did not improve OS, neither in patients with cLNM-negative/pLNM-negative ICC nor in cLNM-negative/pLNM-positive ICC. Therefore, the application of LND for staging and setting postoperative treatment plans may fall to meaninglessness.

The prolonged time span for LND also increases the risk associated with surgery. Kim [29] reported significantly higher risks of postoperative complications in patients who had LND (36.3%, 41/113), compared to those who did not (22.5%, 23/102). The increased postoperative complications included bile leakage, intra-abdominal fluid collection, wound infection, ileus. In the present study, LND group showed longer postoperative hospital stay than that of Non-LND group (9.8 day vs. 8.7 day, p = 0.03). Therefore, LND should be considered with caution during the surgery if it would bring no survival benefit for ICC patients [30].

Other factors in the present study that influenced prognosis were tumor number, tumor size, differentiation, and levels of CA19-9, CEA, and GGT, consisted with literature reports [10, 31, 32]. One of the advantages of our study is the large number of cases enrolled. Most patients (65%) reached the 3-year follow-up. However, we also note the limitations in this study. Our results were based on retrospective data from a single institution. The results need be further validated in prospective, randomized controlled trials.

Different from previous study, this study focused only on the patients with negative evidence of LNM by intraoperative palpation and imaging examination before operation. We discussed the necessity of prophylactic LND in various aspects, including survival benefit, accurate staging and setting postoperative treatment plans. To our knowledge, this is the first study focused on cLNM-negative ICC patients and evaluated the prognostic value of LND in these patients. The results suggested that without sufficient indication, routine LND should not be applied for ICC patients, especially for those evaluated as negative LNM before operation.

## MATERIALS AND METHODS

### Patients

The study protocol was approved by the Clinical Research Ethics Committee of Zhongshan Hospital, Fudan University. ICC in the present study refers to adenocarcinoma arising from second order or more distal branches of the intrahepatic bile ducts. The inclusion criteria are as follows. The patient: 1, received curative resection from January 2009 to December 2014; 2, was diagnosed with ICC by 2 experienced pathologists; 3, had no other malignancies concurrently; 4, had no evidence of LNM by intraoperative palpation and imaging examination before operation; and 5, received no anti-tumor treatment before the surgery. Preoperative imaging was obtained either from computed tomography (CT) or magnetic resonance imaging (MRI). In several cases, positron emission tomography-computed tomography (PET-CT) was also performed.

### Surgical procedures and definitions of parameters

The type of hepatectomy was determined by the location of the lesion, based on Couinaud’s classification of hepatic segments. During the procedure, the lymph node status of all patients was assessed by the chief surgeon. Patients with no evidence of LNM by intraoperative palpation as well as negative for LNM based on the imaging examination were defined as negative for clinical lymph node metastasis (cLNM). The extent of lymphadenectomy in the present study included: 1, lymph nodes located around the hepatoduodenal ligament and the hepatic artery; 2, retropancreatic lymph nodes (for ICC originating in the right hemiliver); and 3, lymph nodes around the cardiac portion of the stomach and along the lesser curvature (in patients with ICC originating in the left hemiliver) [13].

In the present study, tumor-node-metastasis (TNM) staging followed the guidelines of the eighth edition of the American Joint Committee on Cancer/International Union against Cancer. Presence of microscopic vascular invasion or macroscopic tumor thrombus was defined as vessel invasion. Tumor size referred to the maximum tumor diameter and 2 cm was adopted as the cut-off value for patient grouping, according to the Liver Cancer Study Group of Japan [33].

### Follow-up

After resection, all patients had informed follow-up every 3-4 months for the first 2 years and then every 4-6 months for the next year. Clinical information was recorded at visits, including results of blood tests and imaging examination, initially performed using ultrasonography and CT or MRI, if recurrence was suspected. Patients also received telephone follow-up every 6 months. All patients were followed up to December 2016.

### Statistical analysis

Overall survival (OS) time was measured from the date of surgery to the date of death. Recurrence-free survival (RFS) time was calculated from the date of surgery to the date of the first clinically-documented tumor recurrence or metastasis, or to the date of death. Comparison between groups was performed using the χ^2^ or Fisher’s exact tests. The OS and RFS were calculated using the Kaplan–Meier method and the log-rank test was used to assess differences. Cox regression model was adopted for multivariable analysis. All statistical analyses were performed with the SPSS 22.0 (SPSS, Chicago, IL, United States) software package. Statistical significance was defined as p < 0.05.

## SUPPLEMENTARY MATERIALS FIGURE AND TABLE


