# Supplementary material for: Intrahepatic cholangiocarcinoma patients without indications of lymph node metastasis not benefit from lymph node dissection
Source: Oncotarget. 2017 Dec 1;8(69):113817–27. doi: 10.18632/oncotarget.22852 (PMC5768365; doi:10.18632/oncotarget.22852)
Supplement: Supplementary file 1 [file oncotarget-08-113817-s001.pdf]

Intrahepatic cholangiocarcinoma patients without indications of lymph node metastasis not benefit from lymph node dissection

SUPPLEMENTARY MATERIALS

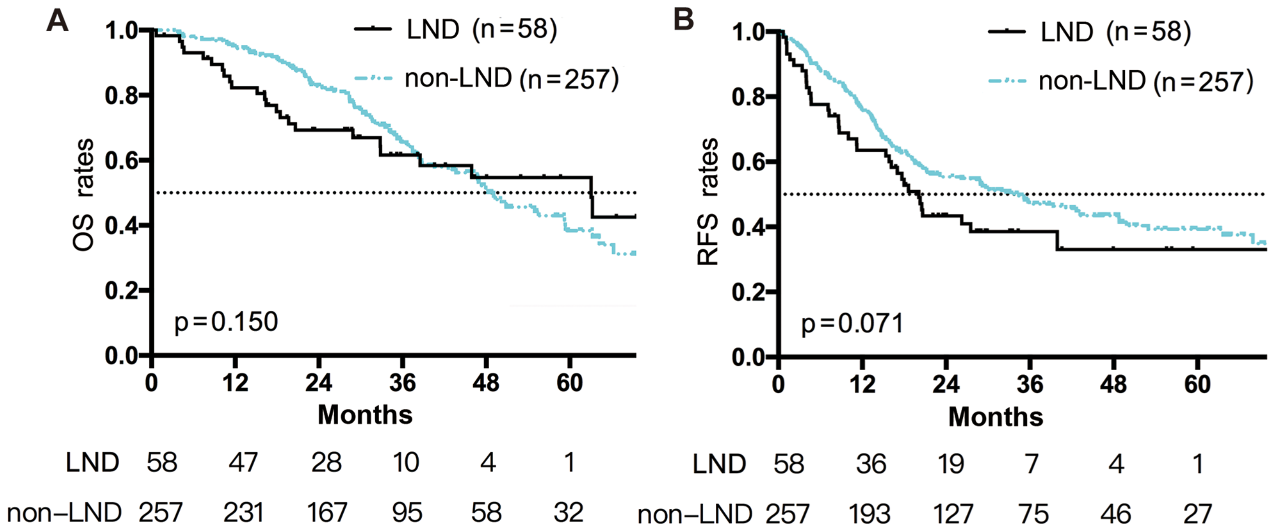

Supplementary Figure 1: OS and RFS curves of ICC patients with solitary tumor and negative vessel invasion. (**A**) OS curves of patients in the lymph node dissection (LND) or non-LND groups. There was no significant survival difference between the two groups (p = 0.15). (**B**) RFS curves of patients in the LND and non-LND groups. There was no significant survival difference between the two groups (p = 0.07). Numbers below the graphs show the number of remaining patients at the time point.

Supplementary Table 1: Clinical characteristics of LND patients

Variable	LND Patients (n = 73)		pLNM- (n = 58)		pLNM+ (n =15)		p
	NO.	%	NO.	%	NO.	%	
**Age, years**							0.55
Median	61		61		60		
Range	40-74		40-74		50-72		
**Sex**							0.73
Male	37	50.7	30	51.7	7	46.7	
Female	36	49.3	28	48.3	8	53.3	
**Tumor number**							0.16
Solitary	62	84.9	51	87.9	11	73.3	
Multiple	11	15.1	7	12.1	4	26.7	
**Tumor size, cm**							0.6
Median	6		5.5		7		
Range	1.5-12.0		1.5-12.0		2.5-10.0		
**Vessel invasion**							0.41
Vascular	1	1.4	1	1.7	0	0	
Biliary	1	1.4	1	1.7	0	0	
vascular & biliary	0	0	0	0	0	0	
**Type of liver resection**							0.94
Hemihepatectomy	49	67.1	38	65.5	11	73.3	
Extended hemihepatectomy	10	13.7	8	15.5	2	13.3	
Central hepatectomy	13	17.8	11	18.9	2	13.3	
Unknown	1	1.4	1	1.7	0	0	
**T stage**							0.69
T1	59	80.8	48	82.8	11	73.3	
T2							
T2a	2	2.7	2	34.4	0	0	
T2b	11	15.1	7	12.1	4	26.7	
T4	1	1.4	1	1.7	0	0	
**Adjunctive treatment**							0.66
Yes	33	45.2	27	46.6	6	39	
No	40	54.8	31	53.4	9	61	
**Postoperative hospital stay, days**							0.97
Average	9.8		8		9		
Range	3-37		4-35		3-37		

LND, Lymph node dissection.
